# Effects of In-House Cryoprecipitate Use on Transfusion Volume for Cardiopulmonary Bypass Surgery

**DOI:** 10.5761/atcs.oa.25-00166

**Published:** 2025-12-09

**Authors:** Wakana Niwa, Yoshiyuki Takami, Atsuo Maekawa, Koji Yamana, Kiyotoshi Akita, Kentaro Amano, Kazuki Matsuhashi, Yasushi Takagi, Tomonobu Abe

**Affiliations:** Department of Cardiac Surgery, Fujita Health University School of Medicine, Toyoake, Aichi, Japan

**Keywords:** cryoprecipitate, cardiopulmonary bypass, blood transfusion, fibrinogen

## Abstract

**Purpose:**

Cryoprecipitate has been covered by Japanese national health insurance since 2020 for cardiopulmonary bypass (CPB)-induced hypofibrinogenemia. This study evaluated the clinical efficacy of in-house cryoprecipitate use in patients undergoing CPB.

**Methods:**

A total of 1357 patients were divided into 2 groups before and after cryoprecipitate introduction in February 2020 (Group A, n = 685; Group B, n = 672). Propensity score matching also compared 205 pairs between transfused patients in Group A (Group A’, n = 597) and those receiving cryoprecipitate in Group B (Group B’, n = 222).

**Results:**

Cryoprecipitate was used in 222 patients (37%) in Group B. While overall transfusion rates did not differ significantly, postoperative red blood cell (20% vs 13%, *p* <0.01) and platelet concentrate (PC) (35% vs 12%, *p* <0.01) use were significantly lower in Group B. In the matched cohorts, including ~70% undergoing aortic surgery, postoperative PC use was significantly reduced in Group B’ (26% vs 18%, *p* = 0.04).

**Conclusions:**

In-house cryoprecipitate use was associated with reduced postoperative PC transfusion, particularly in aortic surgery involving prolonged CPB and deep hypothermic circulatory arrest. A cryoprecipitate-centered hemostatic strategy, supplementing multiple coagulation factors beyond fibrinogen, may be effective in complex CPB procedures.

## Introduction

Postoperative bleeding requiring blood transfusion remains one of the most common complications following cardiovascular surgery under cardiopulmonary bypass (CPB).^1)^ An increased volume of allogeneic blood transfusion, as well as re-exploration for hemostasis, is recognized as a strong independent risk factor for adverse postoperative outcomes, including increased operative mortality, prolonged mechanical ventilation, acute respiratory distress syndrome, sepsis, surgical site infections, and atrial arrhythmias.

The etiologies of bleeding associated with CPB are multifactorial. Factors such as extensive surgical trauma, prolonged blood contact with the artificial surfaces of the extracorporeal circuit, administration of high-dose heparin, and induced hypothermia contribute to the development of thrombocytopenia and coagulopathy.^2)^ Among the various contributors to coagulopathy, a significant hypofibrinogenemia has been identified as a key factor.^3)^ Decreases in fibrinogen levels of 30%–50% are common after CPB.^4)^ Even in the presence of moderate thrombocytopenia, hemostasis can be maintained in cardiac surgical patients if fibrinogen concentrations are ≥200 mg/dL.^5)^ Also, there is an inverse correlation between fibrinogen levels and bleeding risk, even within the normal reference range (150–400 mg/dL). The international guidelines typically recommend the use of cryoprecipitate or fibrinogen concentrate to maintain fibrinogen levels above 1.0 g/L.^6,7)^

For the management of severe hypofibrinogenemia, fresh frozen plasma (FFP) with relatively low concentrations of fibrinogen is not adequate. In the United States and Australia, cryoprecipitate, which contains not only fibrinogen but also other coagulation factors such as factor VIII and von Willebrand factor, is commonly used.^8)^ Conversely, in most European countries, with the exception of the United Kingdom and Canada, fibrinogen concentrates are predominantly used due to their ease of administration and lower perceived risk of viral transmission.^9)^ Despite ongoing debate regarding the comparative efficacy of these two products, fibrinogen concentrates are not currently approved in Japan to treat acquired hypofibrinogenemia associated with CPB, whereas cryoprecipitates have been covered by national health insurance since 2020. Thus, in February 2020, our institution initiated in-hospital cryoprecipitate production for the patients undergoing CPB surgery.

The present study aims to evaluate the clinical efficacy of our in-house cryoprecipitates in patients undergoing CPB surgery, especially by comparing allogeneic blood product transfusion requirements between two patient cohorts: those undergoing CPB surgery during the three years prior to, and those following, the introduction of cryoprecipitate at our institution.

## Materials and Methods

### Study patients

**Fig. 1:**
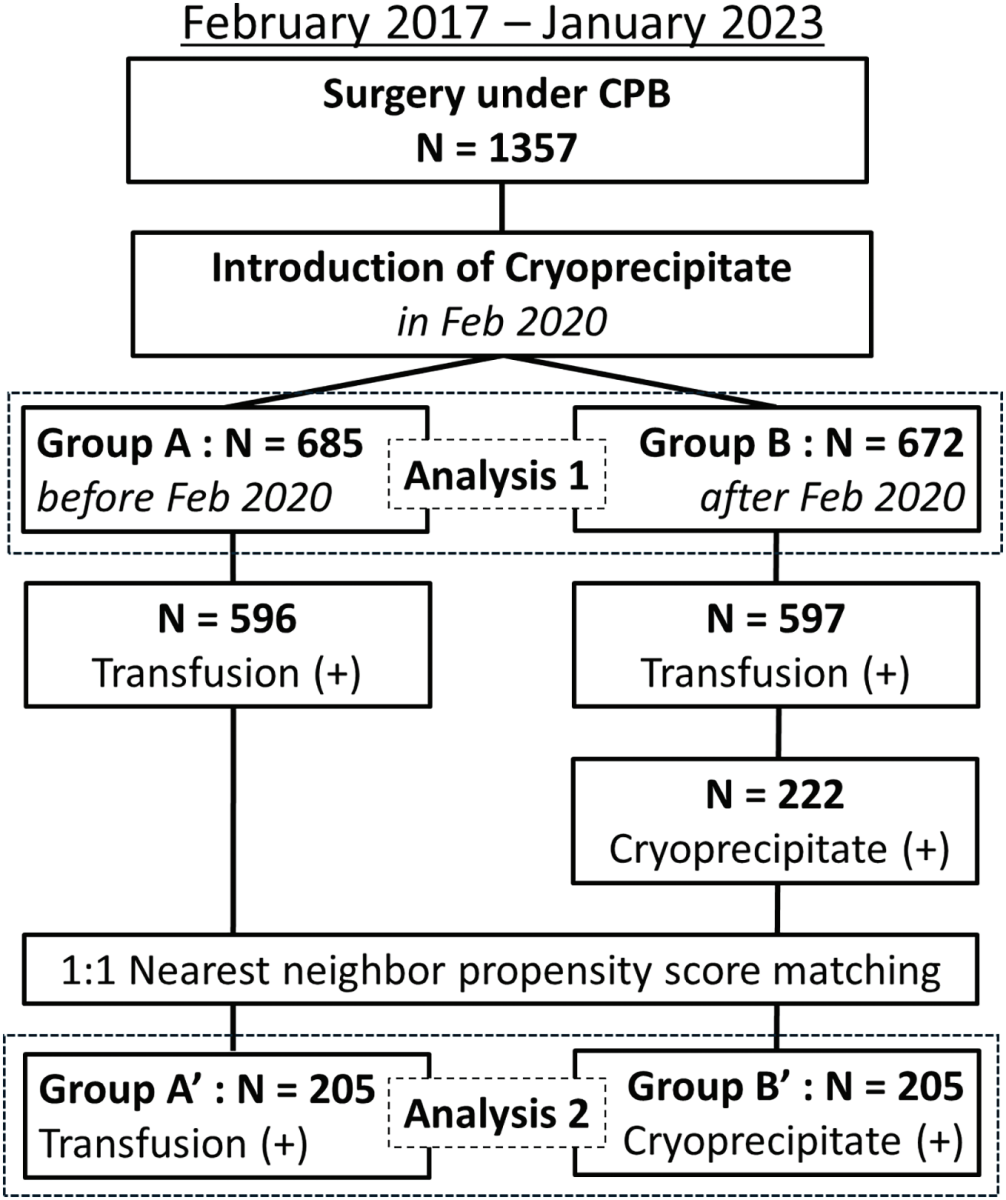
Flowchart showing the inclusion criteria for study patients. CPB: cardiopulmonary bypass

This study included 1357 patients who underwent CPB surgery at Fujita Health University Hospital between February 2017 and January 2023. The patients were divided into two groups based on the introduction of in-hospital cryoprecipitate in February 2020. Group A comprised 685 patients who underwent CPB surgery between January 2017 and January 2020, while Group B included 672 patients who underwent surgery between February 2020 and January 2023 (**Fig. 1**). Patients who underwent off-pump coronary artery bypass grafting or transcatheter aortic valve implantation were excluded from the study.

For CPB, an initial heparin dose of 500 IU/kg was administered, and the activated clotting time (ACT) was measured two minutes later. CPB was commenced when the ACT exceeded 480 seconds. During CPB, the ACT was maintained above 500 seconds on ACT, as measured at 30-minute intervals, by administering 5000 units of heparin. After CPB was weaned, protamine sulfate was administered in equal doses to measure ACT.^10)^

Blood components were transfused according to the guidelines issued by the Ministry of Health, Labor and Welfare in Japan,^11)^ although the decision was ultimately left to the discretion of the attending surgeons, anesthesiologists, and intensive care unit (ICU) physicians. In most patients, the transfusion thresholds were as follows: a hemoglobin level below 8.0 g/dL for red blood cells (RBCs), a fibrinogen level below 150 mg/dL for FFP, and a platelet count below 5.0 × 10^4^/μL for platelet concentrate (PC).

Cryoprecipitate use was planned preoperatively for patients undergoing emergency or urgent surgery, redo surgery, procedures involving deep hypothermic circulatory arrest, or prolonged CPB with complex procedures. Intraoperatively, cryoprecipitate was administered immediately after weaning from CPB and reversal of heparin with protamine, based on severe bleeding tendency accompanied by a fibrinogen level below 150 mg/dL.

Furthermore, to reveal the cryoprecipitate use on transfusion volume in detail, a propensity score matching analysis was performed to compare the data between the patients who received transfusion in Group A and those who received cryoprecipitate in Group B (**Fig. 1**).

### In-house cryoprecipitate

In February 2020, our institution initiated in-hospital cryoprecipitate production in accordance with the method described in the Japan Society of Blood Transfusion and Cell Therapy production protocol.^12)^ Specifically, one pack of cryoprecipitate was prepared from FFP (FFP-LR-480, 480 mL) by collecting the precipitate formed during controlled thawing at 4°C for 24 hours, followed by centrifugation at 4°C at 3000 *g* for 15 minutes, and re-suspending it in 30–50 mL of plasma. Cryoprecipitate can be stored for up to 1 year at a temperature of −40°C or below. A pack of cryoprecipitate prepared from three FFP-LR-480 units was thawed within 9 minutes using a recirculating water-bath thawing system at 37°C (FF-40; Kawasumi Laboratories Inc., Oita, Japan). The fibrinogen level in the in-house cryoprecipitate was 2120 ± 503 mg/dL.

### Data collection

The following information was obtained from the database and electronic medical record system: age; height; weight; sex; comorbidities; chest radiographic, electrocardiographic, and echocardiographic findings; preoperative laboratory variables; operative methods; operative time; CPB time; cardiac ischemic time; minimum body temperature (bladder); total volumes of RBC, FFP, PC, and cryoprecipitate transfused in the operating room and within the first 24 hours after ICU admission; intraoperative blood loss; postoperative drain output; incidences of reexploration for bleeding and deep sternal wound infection; and in-hospital mortality.

Blood products such as RBC, FFP, and PCs were supplied by the Japan Red Cross Society (Tokyo, Japan). The RBC solutions (1 unit: 140 mL; 2 units: 280 mL) and FFP (1 unit: 120 mL; 2 units: 240 mL) were produced from donated whole blood, while the PCs and 480-mL FFP were obtained via apheresis from donors.

### Statistical analysis

All statistical analyses were executed using software (EZR^13)^, available on the website). A *p* value less than 0.05 was deemed significant for all tests. Categorical variables were expressed as counts and percentages and compared using the chi-squared test. Continuous variables were expressed as mean ± standard deviation and compared using analysis of variance or Kruskal–Wallis rank-sum test with appropriate post hoc tests (Tukey’s honestly significant differences or Steel–Dwass, respectively), according to the Kolmogorov–Smirnov test to check for normal distribution.

Propensity score matching was performed to balance risk factors between the groups using 1:1 nearest-neighbor matching with a caliper of 0.1. To assess covariate balance (potential confounders), standardized mean difference was used, and logistic regression was employed for propensity score matching.

## Results

### Comparison of the complete cohort (Group A vs Group B)

The demographic characteristics, examination findings, and laboratory variables of the 2 groups are summarized in **Table 1**. There were no significant differences in any variables, including preoperative hemoglobin value, platelet count, and fibrinogen level, between the two groups. Moreover, there were no significant differences in operative data, as shown in **Table 2**, including the types of main procedures, the frequencies of urgency or emergency, and CPB time. Only 8 patients (1.2%) of Group A and 21 patients (3.1%) in Group B underwent minimally invasive or robotic-assisted cardiac surgery.

**Table 1:** Patient characteristics of the complete cohort

Variables	Group A (n = 685)	Group B (n = 672)	*p* Value
Age (years)	68 ± 11	68 ± 12	0.73
Male gender (n (%))	453 (66)	444 (66)	0.93
Height (cm)	161.8 ± 9.9	161.9 ± 9.6	0.85
Weight (kg)	61.3 ± 13.1	62.0 ± 13.0	0.42
Body mass index (kg/m^2^)	23.3 ± 3.8	23.6 ± 3.9	0.69
Body surface area (m^2^)	1.65 ± 0.20	1.65 ± 0.20	0.94
Hypertension (n (%))	547 (80)	524 (78)	0.73
Hyperlipidemia (n (%))	406 (59)	355 (53)	0.49
Diabetes mellitus (n (%))	240 (35)	194 (29)	0.61
Peripheral artery disease (n (%))	245 (36)	222 (33)	0.56
Smoking habit (n (%))	338 (49)	323 (48)	0.84
Pulmonary disease (n (%))	55 (8)	377 (6)	0.88
Liver disease (n (%))	25 (3.6)	19 (2.9)	0.62
Chronic kidney disease (n (%))	276 (40)	264 (39)	0.89
Hemodialysis-dependent (n (%))	62 (9)	41 (6)	0.23
Previous stroke (n (%))	68 (10)	44 (7)	0.65
Atrial fibrillation (n (%))	90 (13)	75 (11)	0.43
CTR on x-ray (% )	52.4 ± 7.1	51.6 ± 6.8	0.27
Echocardiography			
LVDd (mm)	50.7 ± 8.7	50.3 ± 8.0	0.71
LVDs (mm)	36.1 ± 10.2	35.6 ± 9.2	0.66
LVEF	0.55 ± 0.11	0.55± 0.11	0.98
Hemoglobin (g/dL)	12.9 ± 2.0	12.8 ± 1.9	0.91
Platelets (×10^4^/μL)	20.0 ± 6.4	20.3 ± 6.4	0.82
Fibrinogen (mg/dL)	267 ± 73	272 ± 82	0.84
Serum albumin (g/dL)	3.9 ± 0.5	3.9 ± 0.5	0.94
Total bilirubin (mg/dL)	0.8 ± 0.5	0.7 ± 0.7	0.72
Serum creatinine (mg/dL)	1.64 ± 2.06	1.48 ± 2.00	0.29

Data are presented as mean ± standard deviation or frequency (percentage).

CTR: cardiothoracic ratio; LVDd: left ventricular end-diastolic dimension; LVDs: left ventricular end-systolic dimension; LVEF: left ventricular ejection fraction

**Table 2:** Operative data in the complete cohort

Variables	Group A (n = 685)	Group B (n = 672)	*p* Value
Urgency/emergency	63 (9)	72 (11)	0.25
Redo surgery	38 (6)	34 (5)	0.54
Main procedures			
Ischemic (n (%))	236 (35)	202 (30)	0.21^*^
Valve (n (%))	249 (36)	250 (37)	
Aorta (n (%))	164 (24)	190 (28)	
Others (n (%))	36 (5)	30 (4)	
Concomitant procedures (n (%))	199 (29)	182 (27)	0.76
Under circulatory arrest	146 (21)	162 (24)	0.32
Operation time (min)	408 ± 135	399 ± 128	0.66
CPB duration (min)	194 ± 82	191 ± 79	0.68
Cardiac ischemic time (min)	127 ± 71	131 ± 68	0.47
Lowest bowel temperature (°C)	29.6 ± 5.2	29.6 ± 4.9	0.83

Data are presented as mean ± standard deviation or frequency (percentage).

*Pearson’s chi-squared test.

CPB: cardiopulmonary bypass

**Table 3** shows transfusion data. The blood transfusion rates were similar (87% in Group A vs 89% in Group B, *p* = 0.32). Cryoprecipitate was used in 215 patients (36%) in Group B during surgery and in 7 patients (1.2%) after surgery. There were no significant differences in the used unit and transfusion rate of RBC and PC during surgery between the two groups. Although the transfusion rate of FFP was similar, the volume of transfused unit of FFP was significantly lower in Group B than in Group A. However, there was no difference in the used unit between the two groups, when the used units for cryoprecipitate were taken into account in Group B.

**Table 3:** Clinical and transfusion data of the complete cohort

Variables	Group A (n = 685)		Group B (n = 672)		*p* Value	
Transfusion, n (%)						
No	89 (13)		75 (11)		0.32	
Yes	596 (87)		597 (89)			
Intraoperative	Units	n (%)	Units	n (%)		
RBC (unit)	12.6 ± 6.9	590 (99)	13.3 ± 7.6	595 (99)	0.88	1.00
FFP (unit)	15.7 ± 7.4	585 (98)	14.8 ± 7.6	581 (97)	0.21	0.92
Platelets (unit)	25.6 ± 8.5	295 (49)	25.4 ± 7.7	292 (49)	0.89	0.94
Cryoprecipitate (pack)			1 ± 0	215 (36)		
Postoperative	Unit	n (%)	Unit	n (%)		
RBC (unit)	6.6 ± 9.6	117 (20)	7.8 ± 11.0	75 (13)	0.87	<0.01
FFP (unit)	11.9 ± 13.0	117 (20)	14.1 ± 13.1	98 (16)	0.65	0.58
Platelets (unit)	21.0 ± 9.7	208 (35)	22.1 ± 12.7	70 (12)	0.88	<0.01
Cryoprecipitate (pack)			1 ± 0	7 (1.2)		
Intraoperative blood loss (g)	2732 ± 1201		2536 ± 1043		0.67	
Fibrinogen at ICU arrival (mg/dL)	288 ± 123		294 ± 137		0.74	
24-hour chest tube drainage (mL)	932 ± 521		845 ± 438		0.44	
Reexploration for bleeding (n (%))	11 (1.6)		9 (1.3)		0.82	
Deep sternal wound infection (n (%))	15 (2.2)		13 (1.9)		0.85	
In-hospital death (n (%))	33 (4.8)		24 (3.6)		0.28	

Data are presented as mean ± standard deviation or frequency (percentage).

RBC: red blood cell; FFP: fresh frozen plasma; ICU: intensive care unit

Postoperatively, although there was no significant difference in the use rate of additional FFP administered in ICU, the use of RBCs and PCs was significantly lower in Group B than in Group A. Intraoperative blood loss, fibrinogen level at ICU arrival, 24-hour chest tube drainage, and the incidences of reexploration for bleeding, deep sternal wound infection, and in-hospital death were similar between the two groups.

### Comparison of the matched cohort

The propensity score matching analysis was used to compare data between patients who received a transfusion in Group A (n = 597) and those who received cryoprecipitate in Group B (n = 222). To generate a balanced cohort, Group A′ and Group B′, consisting of 205 pairs, were created. The following covariates, which were significantly different in univariable analysis, were entered into the multivariable models: age, sex, height, dyslipidemia, diabetes mellitus, hemodialysis, emergency surgery, redo surgery, main procedure, and circulatory arrest. After adjusting for baseline characteristics and operative data, as shown in **Tables 4** and **5**, most included patients were those who underwent aortic surgery under circulatory arrest. The matched patients of Group B′ had significantly lower use of post-operative PC in the ICU compared to the matched patients of Group A′, although intraoperative blood product use and postoperative use of RBC and FFP were similar (**Table 6**). There were no significant differences between the two groups in intraoperative blood loss, fibrinogen levels at ICU arrival, 24-hour chest tube drainage, the incidence of reexploration for bleeding and deep sternal wound infection, or in-hospital death.

**Table 4:** Propensity score-matched comparisons of patient characteristics

Variables	Unmatched			Matched		
	Patients with transfusion in Group A	Patients with cryoprecipitate in Group B	*p* Value	Group A′ (patients with transfusion in Group A)	Group B′ (patients with cryoprecipitate in Group B)	*p* Value
n	597	222		205	205	
Age (years)	69 ± 11	68 ± 11	0.09	68 ± 12	68 ± 11	0.79
Male gender (n (%))	374 (63)	142 (65)	0.68	128 (62)	131 (64)	0.84
Height (cm)	161.2 ± 9.9	162.7 ± 9.8	0.08	161.1 ± 9.7	162.1 ± 9.6	0.34
Weight (kg)	69.4 ± 13.2	63.2 ± 14.1	0.67	61.1 ± 13.8	63.0 ± 13.8	0.16
Hypertension (n (%))	469 (79)	162 (73)	0.16	156 (74)	150 (73)	0.88
Hyperlipidemia (n (%))	340 (57)	91 (41)	<0.001	94 (46)	81 (40)	0.23
Diabetes mellitus (n (%))	197 (33)	37 (17)	<0.001	34 (17)	37 (18)	0.79
Peripheral artery disease (n (%))	146 (21)	23 (10)	0.22	29 (14)	22 (11)	0.35
Smoking habit (n (%))	278 (47)	106 (48)	0.69	94 (46)	98 (48)	0.62
Pulmonary disease (n (%))	35 (6)	18 (8)	0.38	12 (6)	18 (8)	0.56
Liver disease (n (%))	23 (4)	8 (4)	1.000	6 (3)	8 (4)	0.74
Chronic kidney disease (n (%))	244 (41)	88 (40)	0.87	82 (40)	8o (39)	0.93
Hemodialysis-dependent (n (%))	62 (10)	11 (5)	0.02	14 (7)	11 (5)	0.68
Previous stroke (n (%))	87 (15)	22 (10)	0.10		22 (10)	0.65
Hemoglobin (g/dL)	12.9 ± 2.0	13.0 ± 2.0	0.87	12.7 ± 2.0	13.0 ± 2.0	0.85
Platelets (×10^4^/μL)	20.0 ± 6.4	19.7 ± 6.1	0.66	19.8 ± 6.4	19.5 ± 6.1	0.78
Fibrinogen (mg/dL)	267 ± 73	275 ± 87	0.77	263 ± 89	274 ± 90	0.71

**Table 5:** Propensity score-matched comparisons of operative data

Variables	Unmatched			Matched		
	Patients with transfusion in Group A	Patients with cryoprecipitate in Group B	*p* Value	Group A′ (patients with transfusion in Group A)	Group B′ (patients with cryoprecipitate in Group B)	*p* Value
n	597	222		205	205	
Urgency/emergency	63 (11)	71 (32)	<0.001	61 (30)	67 (33)	0.59
Redo surgery	38 (6)	26 (12)	0.01	24 (12)	21 (10)	0.75
Main procedures						
Ischemic (n (%))	198 (33)	11 (5)	<0.001^*^	15 (7)	11 (5)	0.61^*^
Valve (n (%))	204 (34)	33 (15)		36 (18)	33 (15)	
Aorta (n (%))	161 (27)	160 (72)		145 (72)	145 (71)	
Others (n (%))	34 (6)	16 (7)		9 (4)	16 (7)	
Concomitant procedures (n (%))	182 (30)	100 (45)	<0.001	76 (37)	88 (43)	0.23
Under circulatory arrest	146 (24)	142 (64)	<0.001	128 (62)	130 (63)	0.92
Operation time (min)	421 ± 135	462 ± 136	<0.001	468 ± 162	459 ± 135	0.21
CPB duration (min)	200 ± 81	243 ± 86	<0.001	240 ± 83	241 ± 86	0.89
Cardiac ischemic time (min)	160 ± 56	155 ± 61	0.28	147 ± 67	154 ± 61	0.30
Lowest bowel temperature (°C)	29.3 ± 5.3	25.2 ± 4.2	<0.001	25.3 ± 4.7	25.3 ± 4.3	0.88

CPB: cardiopulmonary bypass

**Table 6:** Propensity score-matched comparisons of clinical and transfusion data

Variables	Unmatched						Matched					
	Patients with transfusion in Group A		Patients with cryoprecipitate transfusion in Group B		*p* Value		Group A′ (Patients with transfusion in Group A)		Group B′ (Patients with cryoprecipitate in Group B)		*p* Value	
n	597		222				205		205			
Intraoperative	Units	n (%)	Units	n (%)			Units	n (%)	Units	n (%)		
RBC (unit)	12.6 ± 6.9	597 (100)	17.3 ± 9.5	222 (100)	<0.001	1.00	16.1 ± 8.4	205 (100)	17.1 ± 9.6	205 (100)	0.12	0.73
FFP (unit)	15.7 ± 7.4	585 (98)	18.1 ± 7.9	222 (100)	<0.001	0.37	19.9 ± 9.0	205 (100)	18.1 ± 8.0	205 (100)	0.86	0.64
Platelets (unit)	25.6 ± 8.5	295 (49)	27.6 ± 7.5	199 (90)	0.32	<0.001	24.6 ± 12.5	205 (100)	25.0 ± 10.7	202 (90)	0.46	0.25
Postoperative	Units	n (%)	Units	n (%)			Units	n (%)	Units	n (%)		
RBC (unit)	6.6 ± 9.6	117 (20)	9.8 ± 12.8	43 (19)	0.82	0.51	2.0 ± 6.6	41 (20)	1.9 ± 7.0	37 (18)	0.74	0.80
FFP (unit)	11.9 ± 13.0	117 (20)	15.6 ± 14.6	71 (32)	<0.001	<0.001	17.1 ± 11.0	61 (30)	15.0 ± 11.3	62 (30)	0.44	0.59
Platelets (unit)	21.0 ± 9.7	208 (35)	24.5 ± 14.8	39 (18)	<0.001	<0.001	22.1 ± 12.7	54 (26)	19.4 ± 14.8	36 (18)	0.001	0.04
Intraoperative blood loss (g)	2883 ± 1338		2742 ± 1273		0.57		2886 ± 1455		2726 ± 1268		0.59	
Fibrinogen at ICU arrival (mg/dL)	279 ± 165		282 ± 157		0.43		271 ± 176		278 ± 148		0.64	
24-hour chest tube drainage (mL)	1056 ± 722		987 ± 826		0.32		1065 ± 679		992 ± 767		0.48	
Reexploration for bleeding (n (%))	11 (1.8)		5 (2.3)		0.78		5 (2.4)		5 (2.4)		1.00	
Deep sternal wound infection (n (%))	15 (2.5)		5 (2.3)		0.96		7 (3.4)		5 (2.4)		0.77	
In-hospital death	32 (5.3)		16 (7.2)		0.32		13 (6.3)		15 (7.3)		0.85	

RBC: red blood cell; FFP: fresh frozen plasma; ICU: intensive care unit

## Discussion

Our main finding was that intraoperative use of cryoprecipitate reduced PC use after CPB surgery, particularly in patients undergoing aortic surgery with prolonged CPB under deep hypothermic circulatory arrest. According to data from a Japanese institute, three packs of in-house cryoprecipitate provide approximately 2 g of fibrinogen, as each pack reportedly contains 1992 ± 515 mg/dL of fibrinogen.^14)^ We conclude that in-house cryoprecipitate may serve as an effective blood conservation strategy for complex CPB surgeries.

To supplement fibrinogen, which is depleted by CPB and contributes to severe bleeding, either cryoprecipitate or fibrinogen concentrate is recommended.^6,7)^ However, debate continues over which option is more effective for patients undergoing CPB.^9)^ A recent prospective randomized trial comparing cryoprecipitate and fibrinogen concentrate found similar increases in plasma fibrinogen concentrations: 125 ± 65 mg/dL for cryoprecipitate and 96 ± 65 mg/dL for fibrinogen concentrate within 24 hours of administration.^15)^ Thus, distinguishing a clear superiority between the two remains challenging.

Compared to fibrinogen preparations, the disadvantages of cryoprecipitate include the potential for transfusion-related reactions and pathogen transmission due to the lack of pasteurization/viral inactivation, variability in fibrinogen content,^16)^ a required thawing time of 30–45 minutes, and a limited shelf life after thawing of 4–6 hours.^14)^

Conversely, cryoprecipitate offers two important advantages. First, beyond fibrinogen, it contains multiple coagulation factors, including factor VIII, factor XIII, von Willebrand factor, vitronectin, and fibronectin. Factor XIII, also known as the fibrin-stabilizing factor, plays a critical role in hemostasis, and its deficiency is associated with increased postoperative bleeding and reoperation in cardiac surgery.^17)^ Acquired von Willebrand syndrome is frequently observed in patients with prolonged CPB times, those on extracorporeal membrane oxygenation, or those using ventricular assist devices.^18)^ Therefore, the additional coagulation factors in cryoprecipitate may enhance hemostasis in complex cardiac procedures involving prolonged CPB (>200–300 minutes), deep hypothermic circulatory arrest, and significant hemodilution, as demonstrated in our study. Second, cryoprecipitate is considerably more cost-effective than fibrinogen concentrate. A pooled unit of cryoprecipitate costs approximately $300, compared to about $1000 per gram for fibrinogen concentrate in the United States.^19)^

A Japanese survey conducted in December 2021 reported that among 375 facilities, 98 (26%) used fibrinogen concentrate, despite its off-label status in treating acquired hypofibrinogenemia in Japan. In contrast, only 39 facilities (10%) were capable of producing in-house cryoprecipitate,^20)^ despite national health insurance coverage for its use in CPB-related acquired hypofibrinogenemia since 2020. Off-label use is associated with a higher incidence of serious adverse drug reactions and may require substantial pharmacovigilance to detect increased thromboembolic risk. The favorable outcomes associated with cryoprecipitate use in our study may support its broader adoption, offering both clinical and economic advantages.

The present study has several limitations. First, this was a single-center, retrospective observational study with a relatively small sample size. Second, previous studies have demonstrated a significant variation in the fibrinogen content of cryoprecipitate, which ranges from 120 to 796 mg per individual unit.^9,17)^ This variability may lead to an inconsistent hemostatic efficacy for cryoprecipitate. In the present study, the fibrinogen content of the cryoprecipitate administered was not measured; therefore, its effects cannot be considered uniform. Third, in the propensity score matching analysis, the sample size in Group A was reduced from 597 to 205. This substantial reduction raises concerns about potential selection bias and a loss of representativeness in the control group. Fourth, the decision regarding blood transfusion, including cryoprecipitate and PC, was ultimately left to the discretion of the attending surgeons, anesthesiologists, and ICU physicians. As a result, the generalizability of our findings is limited. Fifth, there were no significant differences in FFP use between Group A′ and Group B′. Therefore, there may be a potential risk that the use of cryoprecipitate increases FFP consumption. Finally, although the reduction in postoperative PC transfusion associated with cryoprecipitate use was statistically significant in the propensity-matched cohort, the mean reduction was only three units. To justify the use of cryoprecipitate, high-quality, multicenter randomized controlled trials with larger patient populations are warranted.

## Conclusions

Although the introduction of in-house cryoprecipitate did not demonstrate a clear reduction in intraoperative transfusion volume, its use was associated with decreased postoperative utilization rates and volumes of PC compared to non-use cases, especially in the patients undergoing aortic surgery with longer CPB under deep hypothermic circulatory arrest. Therefore, the hemostatic strategy centered on cryoprecipitate, which contains a broad range of coagulation factors in addition to fibrinogen, may be considered effective.

## Declarations

### Data availability

The datasets analyzed during the current study are available from the corresponding author upon reasonable request.
